# XB130—A Novel Adaptor Protein: Gene, Function, and Roles in Tumorigenesis

**DOI:** 10.1155/2014/903014

**Published:** 2014-06-05

**Authors:** Xiao-Hui Bai, Hae-Ra Cho, Serisha Moodley, Mingyao Liu

**Affiliations:** ^1^Latner Thoracic Surgery Research Laboratories, Toronto General Research Institute, University Health Network, 101 College Street, Toronto, ON, Canada M5G 1L7; ^2^Department of Physiology, Faculty of Medicine, University of Toronto, 1 King's College Circle, Toronto, ON, Canada M5S 1A8; ^3^Institute of Medical Science, Faculty of Medicine, University of Toronto, 1 King's College Circle, Toronto, ON, Canada M5S 1A8; ^4^Department of Surgery, Faculty of Medicine, University of Toronto, 149 College Street, Toronto, ON, Canada M5T 1P5

## Abstract

Several adaptor proteins have previously been shown to play an important role in the promotion of tumourigenesis. XB130 (AFAP1L2) is an adaptor protein involved in many cellular functions, such as cell survival, cell proliferation, migration, and gene and miRNA expression. XB130's functional domains and motifs enable its interaction with a multitude of proteins involved in several different signaling pathways. As a tyrosine kinase substrate, tyrosine phosphorylated XB130 associates with the p85**α** regulatory subunit of phosphoinositol-3-kinase (PI3K) and subsequently affects Akt activity and its downstream signalling. Tumourigenesis studies show that downregulation of XB130 expression by RNAi inhibits tumor growth in mouse xenograft models. Furthermore, XB130 affects tumor oncogenicity by regulating the expression of specific tumour suppressing miRNAs. The expression level and pattern of XB130 has been studied in various human tumors, such as thyroid, esophageal, and gastric cancers, as well as, soft tissue tumors. Studies show the significant effects of XB130 in tumourigenesis and suggest its potential as a diagnostic biomarker and therapeutic target for cancer treatments.

## 1. Introduction

All cellular functions are regulated through various signaling pathways and protein networks. Accumulating evidence suggests that adaptor proteins, which contain multiple protein, lipid, and DNA interaction domains and motifs, play essential roles in mediating signal transduction cascades [1, 2]. Adaptor proteins influence cell proliferation, cell survival, and migration through specific protein-protein and protein-lipid interactions [3]. In this review, we will define the gene and protein structure of the novel adaptor protein, XB130, and illustrate its role in cellular function and human cancers. We hope to draw more attention to this signaling molecule and use it as an example to promote scientific awareness about the importance of adaptor proteins in signal transduction.

## 2. XB130: A Novel Gene and Protein

XB130 was first discovered in our research group and has, subsequently, been studied by several other research groups and scientific consortia to produce a substantial amount of information related to XB130 gene structure, variations, and tissue distribution, which is openly available to the scientific community.

### 2.1. XB130 Gene Information

XB130 was discovered through a molecular cloning process of actin filament associated protein 1 (AFAP1), and its apparent molecular size is of 130 kDa [4]. XB130 is also known as actin filament associated protein 1-like 2 (AFAP1L2) [5], KIAA1914 [6], and PI3KAP [7]. The human* xb130* gene (NCBI gene ID 84632; Unigene cluster Hs.591106; Hugo gene ID 25901; Swiss-Port ID Q8N4X5) is located on chromosome 10 at 10q25.3 between nucleotide positions 116,054,583 and 116,164,515 (http://www.genecards.org/) [8]. Neighboring genes are NHLRC2, TDRD1 (encodes a scaffold protein), VWA2 (involved in cytoskeleton structure stabilization), ADRB1, ABLIM1, and PPIAP19. The proteins, von Willebrand factor A domain containing 2 (VWA2) and actin-binding LIM protein 1 (ABLIM1), are scaffold-like proteins with putative functions in cytoskeleton structural stabilization or remodeling [9, 10]. In addition, at least 10 small nuclear polymorphisms (SNPs) are present in the* xb130* genome (http://www.genecards.org), which may relate to tissue specificity, genetically heritable disease association, or cancer formation and development. The human* xb130* gene has 19 exons that cover the whole coding sequence. Putative homologs of XB130 have been identified from chimpanzee, mouse, rat, bovine, chicken, and zebra fish.

**Figure 1:**
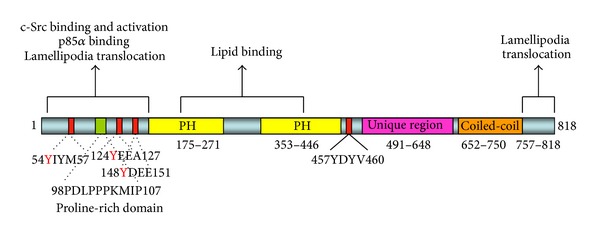
XB130 functional domains and motifs. The N-terminus of XB130 contains a proline-rich SH3 domain binding motif at aa 98–107 and three tyrosine containing SH2 domain binding sites at aa 54–57, aa 124–127, and aa 148–15. The first SH2 domain binding site contains a YXXM motif that binds the SH2 domain in the p85**α** subunit of PI3K. In the middle region, there are two PH domains and another SH2 domain binding site at aa 457–460. The C-terminus contains a coiled-coil region.

The transcript size of human* xb130* is 3,751 bp in length and encodes an 818-amino-acid protein [4]. The molecular weight of this protein by western blot analysis is approximately 130 kDa [4]. Furthermore, there are seven putative splicing variants based on Ensembl sequence alignment ENSG00000169129 [11]. XB130 also contains several tyrosine phosphorylation sites and a proline-rich region (PRR) at the N-terminus, which allows XB130 to interact with Src Homology 2 (SH2) and Src Homology 3 (SH3) domain containing proteins, respectively [4]. In addition, there are two pleckstrin-homology (PH) sequences located in the middle of the protein, which are putative phospholipid and/or membrane binding domains of XB130. Lastly, a coiled-coil domain is located at the C-terminal of XB130, which may be involved in XB130 protein dimerization (Figure 1).

XB130 mRNA is highly expressed in human thyroid and spleen and moderately expressed in other organs, such as brain, pancreas, lung, and kidney [12]. According to the Human Protein Atlas database, XB130 protein is present in thyroid, parathyroid, brain, kidney, skin, and gastrointestinal tracks, which include esophagus, stomach, and colon (http://www.proteinatlas.org/) [13]. RNA sequence analysis by Illumina Body Map also shows expression in lymph node, adipocytes, adrenal gland, breast, ovary, prostate, and testis (http://www.genecard.org/) [8]. The expression of XB130 protein has also been confirmed with immunohistochemistry staining in human thyroid [14], submucosal glands of esophagus [15], and stomach [16].

### 2.2. XB130: A Member of AFAP Family

Like many other discoveries in science, XB130 was discovered serendipitously, while attempting to clone the human AFAP1 gene [4]. Using the chicken AFAP1 cDNA sequence as a search query, XB130 was found in the human EST clone library (GenBank accession 1154093) and showed 40% sequence homology with chicken and human AFAP1. Due to XB130's sequence and structural similarities to AFAP1, XB130 belongs to the AFAP family of proteins.

**Figure 2:**
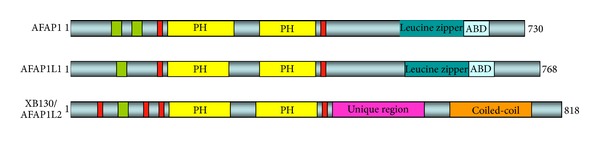
AFAP family proteins. XB130 shares both sequence and structural similarities to the AFAP family of proteins.

The AFAP family contains three members; AFAP1, AFAP1L1, and XB130 (AFAP1L2). All AFAP family members contain two PH domains and a number of SH2 and SH3 binding domain motifs [5]. However, the number and location of SH2 and SH3 binding domain motifs, as well as other unique domains and sequences, differ in each AFAP family member (Figure 2). For example, AFAP1 has two SH3 binding motifs at the N-terminus, while AFAP1L1 and XB130 have only one SH3 binding motif [4, 5, 17]. AFAP1 and AFAP1L1 have one SH2 binding motif, whereas XB130 has three at the N-terminus. The C-termini of AFAP family members bear more functional differences. For instance, AFAP1 and AFAP1L1 contain a leucine zipper and an actin-binding domain (ABD), whereas XB130 only contains a coiled-coil domain in this region (Figure 2) [5]. The unique structural properties and arrangement of these proteins establish their localization in different cellular compartments and determine their binding affinities and interactions with different molecules that are involved in several signaling pathways that influence different cellular functions. For instance, AFAP1 has a strong association with filamentous actin stress fibers involved in cytoskeletal remodeling, focal adhesion formation, and mechanotransduction [17, 18]. AFAP1L1 localizes at the cell periphery in cytoskeletal outgrowths, known as invadosomes, and interacts with the actin polymerization and rearrangement protein, cortactin [5]. XB130, on the other hand, is predominantly localized in the cytoplasm as an adaptor protein for signal transduction and regulates cell proliferation, survival, and gene expression [4, 19]. However, XB130 is also able to translocate to the cell periphery but localizes in cytoskeletal outgrowths known as lamellipodia that function during lateral cell migration [19].

## 3. XB130: A Novel Adaptor Protein in Signal Transduction

Although adaptor proteins lack enzyme catalytic activity, they function by interacting with various molecular partners. XB130 is a good example of an adaptor protein that is able to activate kinases and affect downstream proteins in signaling pathways, leading to changes in cellular function.

### 3.1. XB130 Activates c-Src and Enhances Src-Mediated AP-1/SRE Transcriptional Activation

**Figure 3:**
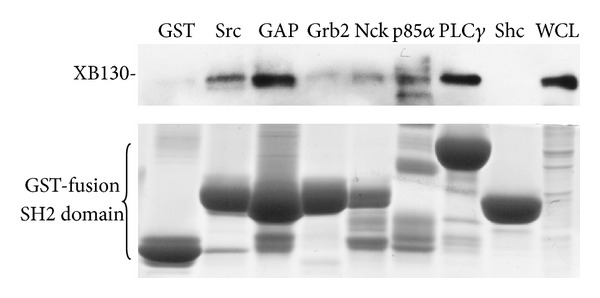
XB130 selectively binds the SH2 domain of several proteins. GST fusion protein pull-down assay shows that XB130 binds to the SH2 domain of Src, GAP, p85**α** subunit of PI3K, and PLC*γ*.

Src protein tyrosine kinases are important signaling molecules that are involved in the regulation of multiple cellular functions [20]. AFAP1 is a known substrate and activator of Src protein tyrosine kinase [17]. Similar to AFAP1, XB130 is also a tyrosine kinase substrate.* In vitro* coexpression of XB130 with a single tyrosine kinase, such as Src [4], RET/PTC [12], Lck [18], and several other protein tyrosine kinases (e.g., EGFR, Abl, and ERBB2), results in XB130 phosphorylation [21]. The XB130-Src interaction was further shown using coimmunoprecipitation (Co-IP) in COS-7 cells that were cotransfected with both XB130 and c-Src. Coexpression of c-Src and XB130 resulted in activation of c-Src both* in vivo* and* in vitro* [4]. XB130 also binds to the SH2 domain of Lck kinase, which is expressed in colorectal cancer cells [22]. Using the GST-fusion protein pull-down assay, it has been shown that XB130 binds SH2 domain containing proteins, such as Src, GAP, p85*α* regulatory subunit of PI3K, and PLC*γ* (Figure 3) [4, 12]. These results indicate that the SH2 and SH3 binding motifs of XB130 are critical for the interaction of XB130 with multiple kinases and may lead to the regulation of cell homeostasis.

Src kinase activity affects cell mitogenesis and transformation by regulating the transcriptional elements, SRE and AP-1 [23–25], as well as the Smad binding element (SBE) involved in TGF-*β* mediated signal transduction [26]. Studies have shown that XB130 activates c-Src and subsequently induces AP-1 and SRE transactivation. However, XB130 interaction with c-Src does not induce Smad-mediated transcriptional activation [4]. The AP-1 transcription factor is known to bind to the promoter region of the chemokine, interleukin-8 (IL-8), which is produced by lung epithelial cells in response to localized infection and inflammation [27]. Introduction of a Src inhibitor or downregulation of XB130 with small interfering RNA (siRNA) reduced IL-8 production in human lung epithelial A549 cells [4]. Consequently, XB130 may be highly involved in inflammation and the innate immune response via the SRE and AP-1 transcriptional elements.

### 3.2. XB130 Binds to the p85*α* Subunit of PI3K and Regulates PI3K Downstream Signaling

Northern blot assay revealed that the highest level of XB130 mRNA is expressed by the thyroid in human [12]. A thyroid specific tyrosine kinase, RET/PTC, is an oncogene due to chromosomal rearrangement, which plays a crucial role in thyroid cancer cells by controlling cell differentiation and proliferation [28]. Co-IP studies show that exogenously overexpressed RET/PTC and XB130 interact in thyroid papillary carcinoma cells. RET/PTC mediates tyrosine phosphorylation of the YxxM motif of XB130 at tyrosine residue 54 [12]. This YxxM motif is the primary binding site of the p85**α** subunit of PI3K. Consequently, RET/PTC promoted the association of the SH2 domain of the p85**α** subunit of PI3K with tyrosine phosphorylated XB130 [12] (Figure 3). Furthermore, knockdown of XB130 in TPC1 papillary thyroid carcinoma cells expressing RET/PTC resulted in reduced Akt phosphorylation at serine residue 473, which blocked cell cycle progression and reduced cell survival [12].

In normal rat thyroid FRTL-5 cells, cyclic adenosine monophosphate (cAMP) treatment increased tyrosine phosphorylation of a 125 kDa protein (p125) and its association with the p85*α* subunit of PI3K [7]. Matrix assisted laser desorption ionization-time of flight (MALDI-TOF) mass spectrometry identified p125 as a rat ortholog of human XB130 (which was labeled as PI3K-associated protein or PI3KAP) [7]. Furthermore, cAMP stimulation increased both XB130 mRNA and protein expression, as well as Src mediated tyrosine phosphorylation of the YxxM motif, which resulted in binding to the p85**α** subunit of PI3K [7]. Inhibition or knockdown of Src kinase abolished cAMP-induced tyrosine phosphorylation of XB130, XB130 interaction with the p85*α* subunit of PI3K, and decreased PI3K activity [7]. In addition, knockdown of XB130 was associated with reduced potentiation of cell synthesis and reduced cyclin D1 protein expression [7]. The evidence presented in this study suggests that XB130 plays an important role in cell cycle progression and proliferation in normal thyroid cells.

These studies demonstrate that Src or other tyrosine kinase mediated-tyrosine phosphorylations of the YxxM motif at the N-terminus of XB130 are a critical event for XB130 binding to the p85*α* subunit of PI3K. The association of XB130 with PI3K is important in the regulation of cell proliferation, cell cycle progression, and cell survival of both normal and cancer cells.

## 4. Cellular Functions of XB130

**Figure 4:**
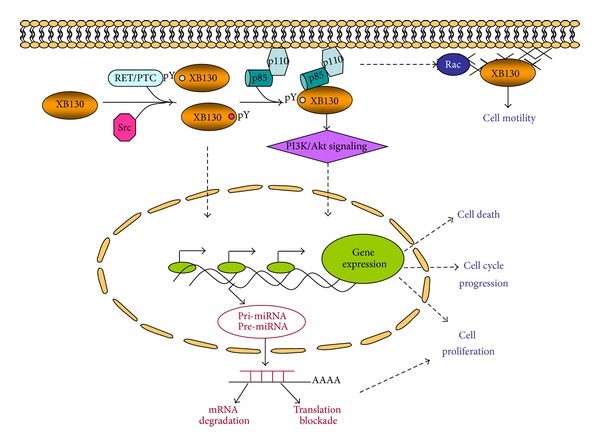
Summary of XB130 cellular functions.

Adaptor proteins are essential in the maintenance of normal cellular physiology and homeostasis by acting as scaffolds and transmitters that bind and translocate molecules to larger complexes or cellular compartments to aid in signal transduction. By acting as an upstream regulator of PI3K, XB130 plays an important role in mediating cell survival and proliferation through Akt pathways [21] and in cell migration through Rac-dependent pathways [12, 19]. The role of XB130 is not limited to protein activity and several studies have shown that XB130 also participates in the regulation of gene expression [14] (Figure 4).

### 4.1. XB130 Modulates Cell Proliferation and Survival

In human thyroid cancer cells, phosphorylated XB130 controls PI3K/Akt activity and subsequently regulates cell proliferation and survival [12]. Other studies have observed similar results using TE2, TE5, and TE9 esophageal squamous carcinoma cells [15], as well as SGC7901 (malignant, metastatic type) and MNK45 (solid tumor under hypoxia type) gastric cancer cell lines [16].

The Akt pathway bears a strong association with cell proliferation and cell survival [29, 30]. To investigate the roles of XB130 in cell survival and proliferation, PI3K/Akt downstream signal pathway proteins were studied in WRO thyroid cancer cells and A549 lung adenocarcinoma cells [21]. The XB130-p85*α* association was detected in both cancer cell lines using Co-IP. Downregulation of XB130 reduced phosphorylation of Akt and the Akt substrate, glycogen synthase kinase *β* (GSK3*β*) [12], which is a serine-threonine kinase that inhibits glycogen synthase assisting in energy metabolism and cell cycle progression [31]. Reduced phosphorylation of Akt resulted in inhibition of Akt activity [12]. Downregulation of XB130 caused a blockade of cell cycle progression from G1 to S phase in both WRO and A549 cells [12]. In addition, measurement of the cell proliferation markers, Ki67 and proliferating cell nuclear antigen (PCNA), showed that knockdown of XB130 reduces cell proliferation [12]. Studies reported that activated Akt induces phosphorylation of p21 Cip/WAF1 (or cyclin dependent kinase interacting protein-1, CDKI-1) and p27 Kip1 (or cyclin dependent kinase inhibitor-1B, CDKI-1B) [21, 32]. Moreover, the accumulation of phosphorylated p21 Cip/WAF1 and p27 Kip1 proteins in the cytoplasm promotes cell survival [32]. RNAi silencing of XB130 reduced phosphorylation of p27 Kip1, p21 Cip/WAF1, and FOXO3a and inhibited cell survival in WRO and A549 cells [21]. These results suggest that XB130 regulates cell proliferation and cell survival by mediating the PI3K/Akt pathway and its downstream proteins, p21 Cip/WAF1, p27 Kip1, and FOXO3a, in thyroid and lung adenocarcinoma cells.

Furthermore, XB130 downregulation in combination with either extrinsic (FasAb) or intrinsic (staurosporine) apoptotic stimuli results in enhanced apoptosis of WRO and A549 cells [21]. The activation of caspase-8 is essential for the progression of extrinsic apoptosis, whereas the activation of caspase-9 is essential for the intrinsic apoptotic pathway [33]. Interestingly, XB130 knockdown cells showed increased levels of cleaved caspase-8 and caspase-9 in WRO cells but decreased procaspase-8 expression and increased caspase-9 cleavage in A549 cells [21]. Other studies suggest that Akt phosphorylates procaspase-9 to prevent its cleavage [34] and that Akt inhibition enhances TRAIL-induced activation of caspase-8 [35]. Therefore, the effects of XB130 on apoptosis or cell death may also be attributed to Akt activity and Akt downstream proteins. However, more experiments are required to better understand this mechanism.

### 4.2. XB130 Controls Expression of Genes Related to Proliferation and Survival

XB130 was previously shown to activate the transcription factors SRE and AP-1, suggesting that XB130 may play a role in the regulation of gene expression. Microarray analysis of XB130 shRNA transfected WRO cells compared to vector transfected control cells demonstrated that XB130 knockdown has a significant effect on gene expression profiles [14]. In total, 57 genes with cell proliferation or survival related function showed a downregulated change in XB130 knockdown cells [36]. Ingenuity pathway analysis listed the top molecular and cellular functions related to XB130 knockdown as cellular growth, proliferation, and cell cycle [36].

MicroRNAs (miRNA, miRs) are a class of small, noncoding RNA molecules regulating gene expression by mediating the degradation of specific target genes [37]. The expression levels of multiple miRNAs were altered in XB130 knockdown WRO cells [36]. The three miRNAs miR-33a, 149a, and 193a-3p, which showed changes in expression level after XB130 downregulation, exhibit tumor suppressive function in thyroid cancer cells [38–40]. The expression of both the pri-miRNA and mature miRNA of miR-33a, 149a, and 193a-3p was increased in XB130 knockdown WRO cells [41]. Overexpression of miR-33a, 149a, and 193a-3p miRNA specific mimics led to protein level reduction of their corresponding targets Myc, SLC7A5, and FOSL1 [41]. Further studies indicated that these miRNAs inhibit their target gene expression by directly binding to the 3′UTR region of the target gene mRNAs causing translation retardation [41]. The regulation of these miRNAs suggests that XB130 is an important mediator at both the gene and protein level.

### 4.3. XB130 Mediates Cytoskeletal Reorganization during Cell Migration

A dynamic cytoskeleton rearrangement is essential for cell motility. The complex nature of cytoskeletal rearrangement and remodeling involves a plethora of molecules. Many adaptor proteins participate in the formation of cytoskeletal outgrowths, such as lamellipodia, membrane ruffles, and podosomes, which are important for cell motility [42–45]. XB130 is also involved in cytoskeleton reorganization.

Although XB130 is known as AFAP1L2, its intracellular distribution is very different from that of AFAP1. As mentioned earlier, AFAP1 and AFAP1L1 contain a well-defined actin-binding motif in their C-termini (Figure 2). The actin-binding motif in AFAP1 is responsible for its binding to F-actin stress fibers and organization of microfilaments [46]. In contrast, XB130 does not possess an actin-binding motif. In BEAS-2B human lung epithelial cells, XB130 is located in the cytoplasm and displays a punctate distribution, whereas AFAP1 appears to colocalize with actin stress fibers [4]. However, in EGF stimulated rat mammary carcinoma (MTLn3) cells, XB130 shows a translocation from cytoplasm to cell periphery [19]. A similar result is observed in phorbol-myristate acetate (PMA) treated TPC1 thyroid cancer cells, in which XB130 appears to translocate to rearranged cytoskeletal structures at the cell periphery [19]. Moreover, when TPC1 cells were transfected with constitutively activated (CA) forms of Rho GTPase, RhoA (Q63L) or Rac1 (Q61L), only the CA-Rac1 expressing cells showed translocation of XB130 to the cell periphery [19]. These motility-induced cells lacked defined central stress fibers but formed lamellipodia at the cell periphery. Interestingly, XB130 translocation did not take place in CA-Rho expressing cells, which maintained strong stress fiber formation. We speculate that XB130's interaction with Rac, which promotes the formation of highly branched F-actin meshwork, results in the association of XB130 to lamellipodia with defined branched F-actin structure. Immunofluorescence (IF) confocal microscopy analysis using cells transfected with different XB130 deletion constructs indicates that both the N-terminus (containing 167aa) and C-terminus (containing 63aa) are required for XB130 movement to the cell periphery [19]. The translocation of XB130 to lamellipodia suggests that XB130 has a putative function in cell migration. Downregulation of XB130 in TPC1 cells results in decreased wound closure area and increased time for wound closure [12], as well as inhibited invasion of cells through a matrigel coated Boyden chamber, reduced lamellipodial persistence, and reduced cell spreading [19]. Thus, XB130 is a novel Rac GTPase-mediated cytoskeleton remodeling protein with high translocation affinity to lamellipodia that contain branched F-actin. Furthermore, XB130 impacts several characteristics of cell migration and invasiveness, such as type and persistence of cytoskeleton structures, cell spreading, and direction of migration, and may prove to be an important feature in tumourigenesis and cancer cell metastasis.

## 5. Clinical Significance of XB130 in Cancer

Deregulation of adaptor proteins is highly related to the abnormality of cellular functions and leads to a spectrum of diseases, including cancer. Many studies have demonstrated that adaptor proteins are also involved in oncogenic signal transduction pathways. For example, the expression of adaptor protein, SH2B1, is elevated in non-small cell lung cancer (NSCLC) tissues [47]. SH2B1's overexpression is considered to be an independent prognostic factor for patients with NSCLC [47]. More interestingly, the adaptor proteins in the AFAP family, AFAP1 and AFAP1L1, are also involved in cancer. AFAP1, for instance, plays a significant role in breast cancer cell adhesion and in the tumorigenesis of prostate cancer [48, 49], whereas AFAP1L1 has been found to be involved in spindle cell sarcomas [50, 51]. Like these AFAP family members, several recent studies indicate that XB130 is also involved in tumorigenesis [36].

### 5.1. XB130 in Human Gastrointestinal Cancers

The first clue that XB130 may be involved in tumorigenesis is from a proteomics study investigating novel biomarkers for colorectal cancers. The Src family member, Lck, was not detectable in normal colonic epithelium but was aberrantly expressed in a subset of colorectal cancer cells [22]. Using mass spectrometry, XB130 was identified as one of the tyrosine phosphorylated proteins that interacts with Lck in colorectal cancer cells, and thus XB130 may be considered as a potential colorectal cancer marker [22]. The oncogenic role of XB130 is also revealed in other gastrointestinal cancers. In normal esophagus, immunohistochemical staining of XB130 protein expression is very low in the epithelium [15]. However, elevated XB130 protein expression was detected in various human esophageal squamous carcinomas [15]. Furthermore, the localization of XB130 in the nucleus of human esophageal squamous carcinoma cells was associated with a shorter 5-year survival rate [15]. Moreover, XB130 protein expression was also reported in gastric cancer tissues. By analyzing gastric cancer samples collected from 411 patients with various stages of cancer, lower protein expression of XB130 in tissue samples was significantly correlated with decreased overall survival time and shorter disease-free period after surgical operation [16]. Thus, it is apparent that XB130 has a major impact on both the progression of human gastrointestinal cancers and the survival of patients.

### 5.2. XB130 in Thyroid Cancers

The first* in vivo* study of XB130 in thyroid cancer tumorigenesis was done by injecting XB130 shRNA stably transfected WRO thyroid cancer cells subcutaneously into nude mice [14]. The volume of tumors formed from XB130 knockdown WRO cells was significantly smaller than those formed from WRO cells transfected with a control vector [14]. The absence of XB130 led to a reduction in tumor growth accompanied with reduced cell proliferation and enhanced apoptosis [14]. A human thyroid tissue-array revealed that XB130 protein is located in the cytoplasm of thyroid follicular cells both in normal thyroid tissue and in papillary thyroid carcinoma [12]. Semiquantitative analysis of cytoplasmic intensity showed that XB130 expression in papillary and anaplastic/insular carcinoma was significantly lower than that in normal tissue and benign lesions [14]. The reduced expression of XB130 in thyroid cancer cells may be due to the disruption of normal tissue structure. In normal thyroid tissue, XB130 is highly expressed and is an essential protein in maintaining normal physiological activity of the thyroid. However, by suppressing XB130 expression, cancer cells may manipulate XB130 mediated signal transduction pathways that control proliferation, survival, and migration to favor tumor growth and invasion.

### 5.3. XB130 in Other Types of Tumors

Soft tissue tumors are a group of neoplasias with different histological and biological features. Gene expression profiling of 102 representative tumor samples varying from benign, locally confined tumors to very aggressive and metastatic tumors revealed that XB130 is one of six highly expressed genes related to local aggressiveness of soft tissue tumors [52]. The gene expression of XB130 was also examined in human hepatocellular carcinoma. Although positive expression of XB130 mRNA was found in 75% of the samples analyzed, the protein expression levels of XB130 in tissue samples were not associated with the prognosis of patients with hepatocellular carcinoma [53]. Based on the Sanger Institute database (http://www.sanger.ac.uk/), XB130 somatic mutations were detected in a variety of tumor samples from lung, large intestine, ovary, skin, prostate, and endometrial tissues. Among these tumors, 70% of the identified cases are caused by substitution missense mutations in the XB130 gene. This new information elucidates an entirely new approach to identify XB130's roles, impact, and tissue specificity in tumorigenesis and cancer progression.

## 6. Prospective

As a newly discovered gene and protein, XB130 has only been studied* in vitro* and* in vivo* by a few research groups. Its functions in the regulation of intracellular signal transduction pathways and gene expression and cellular functions are largely unknown. Thus, our knowledge on XB130* in vivo* is even more limited by the lack of transgenic animals and clinical studies. Further investigation of XB130 and its interacting molecules and related pathways will be fruitful.

### 6.1. Cellular and Molecular Biological Studies

From a cell biology point of view, we are aware that XB130 is an adaptor protein and as such may have many interactors. However, many of its protein and lipid-binding partners are largely unknown. We suggest that yeast-two-hybrid screening and other proteomic approaches should be used to identify XB130's protein-binding partners. Secondly, XB130 contains two PH domains, which are involved in protein-lipid interactions. Exploring the role of XB130 in membrane biology, such as membrane receptors and ion channels, as well as membrane structures related to secretion, endocytosis, and exocytosis, should be considered for future investigation. Finally, the Protein Atlas database shows that XB130 is present in the nuclei of A-431 human epidermoid carcinoma cells, U-2 OS human bone osteosarcoma cells, and U-251 human neuronal glioblastoma cells. Additionally, an immunohistochemical staining of human esophageal squamous carcinoma also revealed XB130 nucleic localization [15]. These sources suggest that XB130 may have potential functions in the nucleus of specific cancer cells.

### 6.2. Physiological Functions of XB130* In Vivo*

Human XB130 mRNA and protein are highly expressed in thyroid and spleen. The thyroid is a major organ that controls body metabolism, energy consumption, and protein synthesis. Dysregulation of XB130 expression in thyroid tissue may result in the dysfunction of the thyroid leading to developmental retardation and hormone disorders. Interestingly, immunohistochemistry staining reveals that XB130 protein expression is high in thyroid follicular cells [12], which are responsible for the synthesis and secretion of thyroid hormones, thyroxine (T4), and triiodothyronine (T3). During thyroid hormone synthesis, numerous proteins are involved in ion transportation across the cell membrane, iodide oxidation, and endocytosis. Thus, XB130, as an adaptor protein, has the potential to be involved in thyroid hormone production, accumulation, and release. The other organ of significance to XB130 expression is the spleen, which is involved in the removal of red blood cells and recycling of iron, as well as the synthesis of antibodies and storage of monocytes [54]. In the spleen, XB130 may play a pivotal role in immune function. As we have seen before, XB130 mediates the release of IL-8, which hints to the role of XB130 in the innate immune response. More impressive is the fact that XB130 is also highly expressed in the epithelial cells of thyroid tissue [14], esophageal submucosal glands [15], and the intestine (http://www.sanger.ac.uk/). Consequently, XB130 may also participate in epithelial cell repair and regeneration by protecting cells from apoptosis and by promoting cell cycle progression and cell migration events in tissues after injury. There are many possible XB130 related tissue-specific mechanisms that are yet to be explored. Future studies of XB130's role in specific tissues and organs may benefit from the use of XB130 knockin or knockout transgenic mice.

### 6.3. XB130 in Tumorigenesis

XB130 expression profiles in multiple cancer tissues reveal that XB130 is involved in tumorigenesis. However, whether it functions as a tumor promoter or a tumor suppressor is still unknown. According to* in vitro* data, XB130 promotes cell proliferation and survival not only by regulating PI3K activity and the Akt pathway, but also by regulating miRNA expression in various cancer cells. MiRNA functional studies are an emerging area of research in tumorigenesis and cancer development and a significant number of miRNAs have been identified as either tumor suppressors or oncogenes [37]. Based on recent findings, we speculate that XB130 affects cancer cell proliferation and survival by modulating the expression of possible tumor suppressing miRNAs and the translation of their target proteins.

XB130 may also directly play a role as a tumor suppressor. Several sources have demonstrated that the XB130 protein expression level is higher in normal tissue or less invasive tumors than in malignant, cancer tissues [14, 52]. Moreover, elevated expression of XB130 was associated with better clinical outcomes in gastric cancer [16]. However, more detailed genetic and epigenetic studies are required to elucidate XB130's roles in different tissue types of tumors and at different stages of cancer progression.

The significant effects of XB130 in tumorigenesis and during cancer development in different tissues support its potential as a diagnostic biomarker and therapeutic target. Using both cell biology techniques and animal models, the roles of XB130 should be further investigated to verify XB130's potential as a diagnostic marker in the prevention of cancer and as a therapeutic target for the continued survival of cancer patients. These studies will also elucidate the molecular mechanisms mediated by the novel adaptor protein, XB130.

## References

[1] Wallez Y, Mace PD, Pasquale EB, Riedl SJ (2012). NSP-CAS protein complexes: emerging signaling modules in cancer. *Genes and Cancer*.

[2] Gery S, Koeffler HP (2013). Role of the adaptor protein LNK in normal and malignant hematopoiesis. *Oncogene*.

[3] Pawson T, Scott JD (1997). Signaling through scaffold, anchoring, and adaptor proteins. *Science*.

[4] Xu J, Bai X-H, Lodyga M (2007). XB130, a novel adaptor protein for signal transduction. *The Journal of Biological Chemistry*.

[5] Snyder BN, Cho Y, Qian Y, Coad JE, Flynn DC, Cunnick JM (2011). AFAP1L1 is a novel adaptor protein of the AFAP family that interacts with cortactin and localizes to invadosomes. *European Journal of Cell Biology*.

[6] Nagase T, Kikuno R, Ohara O (2001). Prediction of the coding sequences of unidentified human genes. XXI. The complete sequences of 60 new cDNA clones from brain which code for large proteins. *DNA Research*.

[7] Yamanaka D, Akama T, Fukushima T (2012). Phosphatidylinositol 3-kinase-binding protein, PI3KAP/XB130, is required for cAMP-induced amplification of IGF mitogenic activity in FRTL-5 thyroid cells. *Molecular Endocrinology*.

[8] Stelzer G, Dalah I, Stein TI (2011). In-silico human genomics with GeneCards. *Human Genomics*.

[9] Gebauer JM, Müller S, Hanisch F-G, Paulsson M, Wagener R (2008). O-Glucosylation and O-fucosylation occur together in close proximity on the first epidermal growth factor repeat of AMACO (VWA2 protein). *The Journal of Biological Chemistry*.

[10] Kim AC, Peters LL, Knoll JHM (1997). Limatin (LIMAB1), an actin-binding LIM protein, maps to mouse chromosome 19 and human chromosome 10q25, a region frequently deleted in human cancers. *Genomics*.

[11] Flicek P, Amode MR, Barrell D (2014). Ensembl 2014. *Nucleic Acids Research*.

[12] Lodyga M, de Falco V, Bai X-H (2009). XB130, a tissue-specific adaptor protein that couples the RET/PTC oncogenic kinase to PI 3-kinase pathway. *Oncogene*.

[13] Uhlen M, Oksvold P, Fagerberg L (2010). Towards a knowledge-based human protein atlas. *Nature Biotechnology*.

[14] Shiozaki A, Lodyga M, Bai X-H (2011). XB130, a novel adaptor protein, promotes thyroid tumor growth. *American Journal of Pathology*.

[15] Shiozaki A, Kosuga T, Ichikawa D (2013). XB130 as an independent prognostic factor in human esophageal squamous cell carcinoma. *Annals of Surgical Oncology*.

[16] Shi M, Huang W, Lin L (2012). Silencing of XB130 is associated with both the prognosis and chemosensitivity of gastric cancer. *PLoS ONE*.

[17] Baisden JM, Qian Y, Zot HM, Flynn DC (2001). The actin filament-associated protein AFAP-110 is an adaptor protein that modulates changes in actin filament integrity. *Oncogene*.

[18] Han B, Bai X-H, Lodyga M (2004). Conversion of mechanical force into biochemical signaling. *The Journal of Biological Chemistry*.

[19] Lodyga M, Bai X-H, Kapus A, Liu M (2010). Adaptor protein XB130 is a Rac-controlled component of lamellipodia that regulates cell motility and invasion. *Journal of Cell Science*.

[20] Okutani D, Lodyga M, Han B, Liu M (2006). Src protein tyrosine kinase family and acute inflammatory responses. *American Journal of Physiology—Lung Cellular and Molecular Physiology*.

[21] Shiozaki A, Shen-Tu G, Bai X (2012). XB130 mediates cancer cell proliferation and survival through multiple signaling events downstream of Akt. *PLoS ONE*.

[22] Emaduddin M, Edelmann MJ, Kessler BM, Feller SM (2008). Odin (ANKS1A) is a Src family kinase target in colorectal cancer cells. *Cell Communication and Signaling*.

[23] Courtneidge SA (2002). Role of Src in signal transduction pathways. The Jubilee Lecture. *Biochemical Society Transactions*.

[24] Boureux A, Furstoss O, Simon V, Roche S (2005). Abl tyrosine kinase regulates a Rac/JNK and a Rac/Nox pathway for DNA synthesis and Myc expression induced by growth factors. *Journal of Cell Science*.

[25] Martin GS (2001). The hunting of the Src. *Nature Reviews Molecular Cell Biology*.

[26] Kim J-T, Joo C-K (2002). Involvement of cell-cell interactions in the rapid stimulation of Cas tyrosine phosphorylation and Src kinase activity by transforming growth factor-*β*1. *The Journal of Biological Chemistry*.

[27] Khanjani S, Terzidou V, Johnson MR, Bennett PR (2012). NF*κ*B and AP-1 drive human myometrial IL8 expression. *Mediators of Inflammation*.

[28] Kim Y-R, Sun HS, Won M (2008). Modulatory role of phospholipase D in the activation of signal transducer and activator of transcription (STAT)-3 by thyroid oncogenic kinase RET/PTC. *BMC Cancer*.

[29] Saini KS, Loi S, de Azambuja E (2013). Targeting the PI3K/AKT/mTOR and Raf/MEK/ERK pathways in the treatment of breast cancer. *Cancer Treatment Reviews*.

[30] Ma J, Sawai H, Matsuo Y (2010). IGF-1 mediates PTEN suppression and enhances cell invasion and proliferation via activation of the IGF-1/PI3K/Akt signaling pathway in pancreatic cancer cells. *Journal of Surgical Research*.

[31] Han B, Xiao H, Xu J (2011). Actin filament associated protein mediates c-Src related SRE/AP-1 transcriptional activation. *FEBS Letters*.

[32] Ping B, He X, Xia W (2006). Cytoplasmic expression of p21CIP1/WAF1 is correlated with IKK*β* overexpression in human breast cancers. *International Journal of Oncology*.

[33] Tang PS, Mura M, Seth R, Liu M (2008). Acute lung injury and cell death: how many ways can cells die?. *American Journal of Physiology—Lung Cellular and Molecular Physiology*.

[34] Cardone MH, Roy N, Stennicke HR (1998). Regulation of cell death protease caspase-9 by phosphorylation. *Science*.

[35] Rokhlin OW, Guseva NV, Tagiyev AF, Glover RA, Cohen MB (2002). Caspase-8 activation is necessary but not sufficient for tumor necrosis factor-related apoptosis-inducing ligand (TRAIL)-mediated apoptosis in the prostatic carcinoma cell line LNCaP. *Prostate*.

[36] Shiozaki A, Liu M (2011). Roles of XB130, a novel adaptor protein, in cancer. *Journal of Clinical Bioinformatics*.

[37] Zhang B, Pan X, Cobb GP, Anderson TA (2007). microRNAs as oncogenes and tumor suppressors. *Developmental Biology*.

[38] Thomas M, Lange-Grünweller K, Weirauch U (2012). The proto-oncogene Pim-1 is a target of miR-33a. *Oncogene*.

[39] Liu H, Brannon AR, Reddy AR (2010). Identifying mRNA targets of microRNA dysregulated in cancer: with application to clear cell renal cell carcinoma. *BMC Systems Biology*.

[40] Uhlmann S, Mannsperger H, Zhang JD (2012). Global microRNA level regulation of EGFR-driven cell-cycle protein network in breast cancer. *Molecular Systems Biology*.

[41] Takeshita H, Shiozaki A, Bai X-H (2013). XB130, a new adaptor protein, regulates expression of tumor suppressive microRNAs in cancer cells. *PLoS ONE*.

[42] Hossain S, Dubielecka PM, Sikorski AF, Birge RB, Kotula L (2012). Crk and ABI1: binary molecular switches that regulate abl tyrosine kinase and signaling to the cytoskeleton. *Genes and Cancer*.

[43] Pauker MH, Reicher B, Fried S, Perl O, Barda-Saad M (2011). Functional cooperation between the proteins Nck and ADAP is fundamental for actin reorganization. *Molecular and Cellular Biology*.

[44] Rider L, Diakonova M (2011). Adapter protein sh2b1*β* binds filamin A to regulate prolactin-dependent cytoskeletal reorganization and cell motility. *Molecular Endocrinology*.

[45] Lányi Á, Baráth M, Péterfi Z (2011). The homolog of the five SH3-domain protein (HOFI/SH3PXD2B) regulates lamellipodia formation and cell spreading. *PLoS ONE*.

[46] Xiao H, Han B, Lodyga M, Bai X-H, Wang Y, Liu M (2012). The actin-binding domain of actin filament-associated protein (AFAP) is involved in the regulation of cytoskeletal structure. *Cellular and Molecular Life Sciences*.

[47] Zhang H, Duan C-J, Chen W (2012). Clinical significance of SH2B1 adaptor protein expression in non-small cell lung cancer. *Asian Pacific Journal of Cancer Prevention*.

[48] Zhang J, Serk IP, Artime MC (2007). AFAP-110 is overexpressed in prostate cancer and contributes to tumorigenic growth by regulating focal contacts. *The Journal of Clinical Investigation*.

[49] Dorfleutner A, Stehlik C, Zhang J, Gallick GE, Flynn DC (2007). AFAP-110 is required for actin stress fiber formation and cell adhesion in MDA-MB-231 breast cancer cells. *Journal of Cellular Physiology*.

[50] Furu M, Kajita Y, Nagayama S (2011). Identification of AFAP1L1 as a prognostic marker for spindle cell sarcomas. *Oncogene*.

[51] Kajita Y, Kato T, Tamaki S (2013). The transcription factor Sp3 regulates the expression of a metastasis-related marker of sarcoma, actin filament-associated protein 1-like 1 (AFAP1L1). *PLoS ONE*.

[52] Cunha IW, Carvalho KC, Martins WK (2010). Identification of genes associated with local aggressiveness and metastatic behavior in soft tissue tumors. *Translational Oncology*.

[53] Zuo Q, Huang H, Shi M (2012). Multivariate analysis of several molecular markers and clinicopathological features in postoperative prognosis of hepatocellular carcinoma. *Anatomical Record*.

[54] Mebius RE, Kraal G (2005). Structure and function of the spleen. *Nature Reviews Immunology*.

